# New York Letter

**Published:** 1898-01

**Authors:** Ogden C. Ludlow

**Affiliations:** 2309 Seventh Avenue; New York


### *CORRESPONDENCE.*

### NEW YORK LETTER.

NEW YORK, December 15, 1897.

The fact that, like the poor, cases of typhoid fever are always with us, probably explains the degree of interest, even approaching enthusiasm, evoked by the announcement that typhoid fever has been selected as the theme for discussion at a medical meeting. This spirit of interest reaches even to those *fin de siècle* practitioners who ordinarily look with disdain on "general medicine" as not being an up-to-date specialty; and they will even admit, on these occasions, that there are a few topics worthy of attention outside of the domain of surgery and gynecology. Certainly, such an opinion finds ample justification; for one who listens to a general discussion on the treatment of typhoid fever cannot fail to be impressed with the great diversity of opinion and the almost total lack of guiding principles that have become crystallized in the practice of the medical fathers. Thus, one practitioner, of many years and large experience with hydrotherapy, will declare, unreservedly, his abiding faith in the Brandt method of treatment, and will be ably seconded by a younger but equally enthusiastic professional brother, who confidently asserts that although a few years ago many of our leading physicians were opposed to this treatment, hardly any can be found now who are willing to oppose it. Another speaker will admit that the "tub-bath treatment is the best routine method in hospital," but adds, that he is not sure that it has, in itself, been the means of saving many lives. Still another will express his entire disapproval of tub-bathing as a *method* of treatment, and will insist that, both at the bedside and in the post-mortem room, he has observed disastrous results from its employment. After swords have been crossed and honors divided over the matter of the bath treatment, the impartial listener and earnest seeker after the truth is likely to be treated to a sharp tilt between the advocates and opponents of intestinal antisepsis, as a prominent feature of the management of typhoid fever. After these important questions have been disposed of for the time being, there usually follow a scattering fire and a good deal of skirmishing over the advisability of using alcoholic stimulants, the coal-tar antipyretics, opiates and cathartics.

The foregoing is not a mere fanciful description, but is a picture drawn from real life, and is intended to introduce and, to some extent, explain what follows.

In a paper on methods of typhoid bathing, based on analysis of 200 cases of typhoid fever observed in hospital practice, Dr. J. P. Thornley stated that of forty-eight patients treated by the tub-bath alone, three died, giving a mortality of 6.2 per cent. Relapses and complications were observed in 37.5 per cent. Twenty-six patients received sponge baths, and the mortality was 15.3 per cent. Of the fifty-eight who received both sponge and tub-baths, 13.7 per cent. died, and relapses occurred in 55.1 per cent. Of seventeen who received neither sponge nor tub-baths, three died, and these three were really moribund on admission to the hospital. The cases treated by both forms of bathing were of a very severe type, while the others were comparatively mild. In discussing this paper, Dr. William H. Thomson said, that out of sixty-one typhoid fever patients treated by him in the Roosevelt Hospital in four months, only five died, and only eleven had delirium. He attributed these good results to the treatment, which consisted in giving a bath as often as necessary to keep the temperature below 103° F., a purgative dose of calomel and jalap twice a week, large doses of saccharated pepsin and bismuth and a diet of equal parts of milk and lime water. Dr. S. Baruch, who is an ardent advocate of the Brandt treatment, said that as the diagnosis could not be positively made before the fifth day, it should be made the rule to begin bathing all suspicious cases early, and without waiting for a positive diagnosis. He followed Brandt's rule of giving a bath at 65° F., every three hours if the rectal temperature was 102° F. or over, and paid no attention to the patient's protests; but if the chattering of the teeth showed that the patient was really too cold, the bath was discontinued. In discussing the same subject on another occasion, Dr. S. S. Burt stated that he very seldom resorted to cold baths, because he had come to the conclusion that while they lowered the surface temperature and stopped heat radiation, the generation of heat still went on, and consequently, after a short time the body temperature was likely to be as high, if not higher than before the bath. Dr. William Henry Porter said that he had abandoned the cold bath treatment after seeing the bath powerless to reduce the temperature below 105° F., and after the autopsy had shown in this case a remarkable degree of congestion of the kidneys. In his opinion, the whole treatment of this disease consisted in decreasing toxin absorption and stimulating the glandular organs to activity. So much for some of the opinions regarding this oft-disputed part of the management of typhoid fever.

At one of the meetings of the Society of Alumni of Bellevue Hospital, Dr. Condict W. Cutler took up the other aspects of this subject. Of the one hundred cases forming the basis of his paper, seventy had been treated by the heroic methods now largely in vogue, and thirty by a less energetic plan. In brief, this latter treatment consisted in the use of calomel and quinine at the very outset, and subsequently in the free use of morphine to quiet the nervous system, and of small doses of whisky to *anticipate* vital depression. The smallest daily dose of morphine that he had used had been one-fifth of a grain, and the largest, two grains. Delirium was quieted by it, and the action of the heart improved, but the drug was contraindicated if there were coma or much stupor already present. High temperature he controlled by sponging with tepid water, followed by frictions. The patient was kept on a diet of peptonized milk until convalescence was assured. In the discussion of this paper, Dr. A. Alexander Smith stated that he was in the habit of controlling marked restlessness by the use of some preparation of opium, or before the eighth day, by combining an opiate with small doses of one of the synthetic coal-tar preparations. He had come to rely almost exclusively on morphine for the control of intestinal hemorrhage, to the exclusion of the ice-bag, which he suspected was sometimes responsible for the recurrence of the hemorrhage. The great danger in this disease is not from the pyrexia, but from the depression of the vital forces, and therefore, it seems probable that physicians have made a mistake in becoming such slaves to the clinical thermometer. The body temperature is not in itself a trustworthy guide to the use of baths; the physician should take into consideration the condition of the nervous and circulatory systems, and of the skin, and in private practice at least, the "bed bath" will be found the most convenient and effective. In the opinion of the writer, Dr. Smith performed an important duty when he uttered a warning against the tendency of the medical profession to adopt a too meddlesome plan of treatment. He clinched the argument by citing cases illustrative of what Nature could do in the way of restoring the sick one to health, even under seemingly very adverse circumstances, when not interfered with in her beneficent work by the injudicious efforts of the physician. In continuing the discussion, Dr. Egbert Le Fevre, said that in studying the pathological findings, one must be impressed with the fact that this is normally a disease of two weeks' duration, and that accordingly our treatment during these first two weeks is of vital importance. He favored at the outset an eliminative treatment, consisting in the administration of small doses of calomel and of large quantities of water. A case was often made severe by giving too much food. Dr. W. H. Katzenbach also emphasized the importance of the early treatment, by stating that the prognosis depended largely upon absolute quiet and good nursing during the first week of the disease. Unless the physician took pains to secure for his patient perfect quiet, both of body and mind, he had not done his whole duty in this very important respect. High temperature could be safely and conveniently controlled by a warm pack, or by sponging with tepid water. In giving such a sponge-bath the nurse often made the mistake of giving it under the bed-clothes, whereas unless the patient were freely exposed to the air during the bath it was almost valueless.

It is evident from the views here expressed, that medical practitioners in this city are far from being of one mind in regard to what constitutes the most approved treatment of this very important disease. It is rather difficult to reconcile the statement, that the routine administration of opiates is highly important, with that which claims that the most successful and rational treatment of typhoid fever to-day is essentially an eliminative one. I may here say, that we do not hear as much now as formerly about the great value of intestinal antiseptics. Some of the most earnest advocates of this treatment have become tired of waging war with these inadequate weapons against the hordes of microbes, and are now content to use the "eliminative treatment," or such other measures as tend to promote intestinal asepsis. Finally, it must be obvious to all that the claim, already quoted, that but few physicians in New York City are now opposed to the cold-bath treatment of typhoid fever, is preposterous.

In an address delivered here by Dr. William Osler, he said that the main points of differentiation between malaria and typhoid fever were the following: In malaria, the onset is much more abrupt, the chills far more frequent, and the initial rise of temperature much more rapid; herpes is exceedingly rare in typhoid fever, but very common in malaria; early delirium is more frequent in malaria; the remittent type of fever is not found in typhoid fever. Although it is quite commonly believed by physicians that typhoid fever not infrequently takes on a malarial aspect, Dr. Osler says that out of five hundred cases of typhoid fever and one thousand cases of malaria, he has only met with one instance, and that a doubtful one, of double infection. Although many of these five hundred typhoid fever patients came from a malarial district, they presented no unusual variations from the accepted type. From his experience with malaria in the South, Dr. S. Baruch is inclined to believe that the diagnosis can usually be made without much difficulty, and that it rests mainly on the fact that the initial variations of temperature are greater than in any other common disease. In his experience, splenic enlargement has been so infrequent as to be of no diagnostic importance. Dr. Osler took exception to this last statement. He added that an important diagnostic point in connection with the temperature was, that in simple intermittent fever, the time from the rising of the temperature above 99° F to the time of its fall below this point, is almost invariably eleven or twelve hours.

A curious method of malarial infection, not very generally known to physicians, was referred to by Dr. J. R. Taylor, of Sag Harbor, Long Island, at a meeting of the Section on General Medicine. He reported several cases of well-marked malaria, in which the diagnosis had been established by the finding of the malarial plasmodia in the blood, which resisted approved methods of treatment until he had insisted that the potted plants be removed from the living-rooms. He had also found the plasmodia in the blood of four florists who had not been exposed to the usual sources of infection. Dr. R. C. Newton, of Montclair, said that he had also seen several instances of this kind.

OGDEN C. LUDLOW, M.D.,

*2309 Seventh Avenue.*

